# The importance of collecting structured clinical information on multiple sclerosis

**DOI:** 10.1186/s12916-016-0627-1

**Published:** 2016-05-31

**Authors:** Tjalf Ziemssen, Jan Hillert, Helmut Butzkueven

**Affiliations:** Center of Clinical Neuroscience, Department of Neurology, MS Center Dresden, Center of Clinical Neuroscience, University Hospital Carl Gustav Carus, Dresden University of Technology, Fetscherstr. 74, 01307 Dresden, Germany; Department of Clinical Neuroscience and Center for Molecular Medicine, Karolinska Institute, Stockholm, Sweden; Department of Neurology, Royal Melbourne Hospital, Victoria, Australia

**Keywords:** Multiple sclerosis, Real-world evidence, Real-world data, Randomised controlled trials, Registries, Pharmacoeconomics

## Abstract

**Background:**

Randomized controlled trials (RCTs) are the ‘gold standard’ in the generation of drug efficacy and safety evidence. However, enrolment criteria, timelines and atypical comparators of RCTs limit their relevance to standard clinical practice.

**Discussion:**

Real-world data (RWD) provide longitudinal information on the comparative effectiveness and tolerability of drugs, as well as their impact on resource use, medical costs, and pharmacoeconomic and patient-reported outcomes. This is particularly important in multiple sclerosis (MS), where economic treatment benefits of long-term disability reduction are a cornerstone of payer drug approvals – these are typically not examined in the RCT itself but modelled using real-world datasets. Importantly, surrogate markers used in RCTs to predict the prevention of long-term disability progression can only truly be assessed through RWD methodologies.

**Summary:**

We discuss the differences between RCTs and RWD studies, describe how RWD complements the evidence base from RCTs in MS, summarize the different methods of RWD collection, and explain the importance of structuring data analysis to avoid bias. Guidance on performing and identifying high-quality real-world evidence studies is also provided.

## An introduction to real-world evidence (RWE) in multiple sclerosis (MS)

**Fig. 1:**
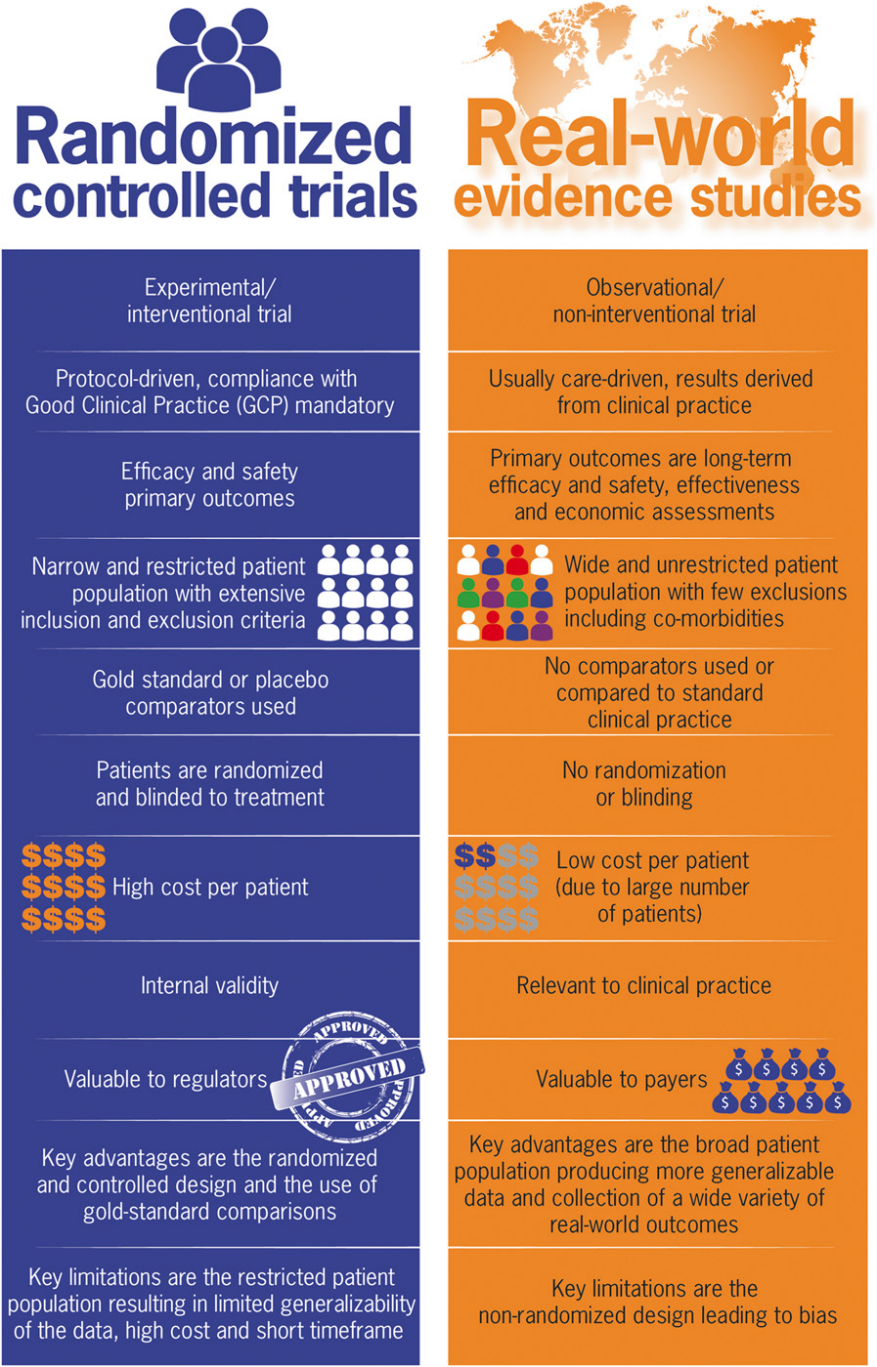
The key differences between randomized controlled trials and real-world evidence studies [1, 3, 4]

In general, randomised controlled trials (RCTs) and RWE studies are important for improving our understanding of disease outcomes and treatment effects, with the two methodologies being increasingly viewed as complementary by clinicians, the pharmaceutical industry, drug regulatory and reimbursement agencies, and patients [1]. The prominence and value of RWE studies (Box 1) have made them mandatory in many settings; however, inherent variability in patient care means that their analysis requires particular care. Figure 1 summarises the key differences between RCTs and RWE studies.

**Fig. 2:**
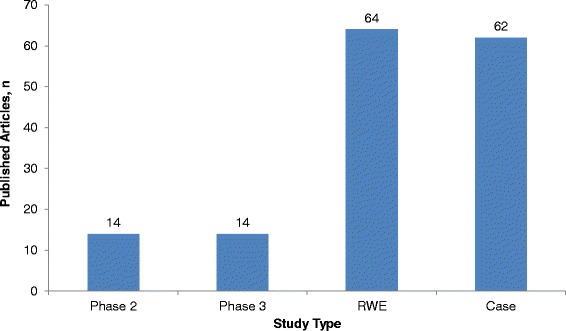
Number of multiple sclerosis (MS) articles of different study types published in 2014. Numbers of each type of study were found by searching the PubMed database for articles published between January 1, 2014, and December 31, 2014, using the following search terms: for phase 2 clinical trials: (‘multiple sclerosis’ OR ‘MS’) AND (‘Phase II clinical trials’ OR ‘Phase 2 clinical trials’); for phase 3 clinical trials: (‘multiple sclerosis’ OR ‘MS’) AND (‘Phase III clinical trials’ OR ‘Phase 3 clinical trials’); For real-world evidence studies: (‘multiple sclerosis’ OR ‘MS’) AND (‘real world’ OR ‘comparative effectiveness’ OR ‘registry’); for case studies: (‘multiple sclerosis’ OR ‘MS’) AND (‘case study’)

In MS, there is a current and growing emphasis on obtaining data beyond phase 3 RCTs. In 2014, the number of published RWE studies in MS exceeded that of published phase 2 and 3 studies by more than two-fold (Fig. 2). This has been driven by increasing demand from payers and healthcare decision-makers for post-approval evidence to inform reviews of pricing, reimbursement, licences for new therapies, and formulation and indication changes [2]. Post-approval RWE studies are important for providing information on compliance with current treatment guidelines, identifying suboptimal therapies, defining treatment responder subgroups, optimizing treatment sequencing, and monitoring rare serious adverse events. This information can support licence extensions and treatment sequencing [3].

RWE studies can also provide valuable insights prior to product development. Pre-launch RWE studies, for example, are useful for mapping out the natural and drug-modified history of disease, current practice patterns and service structures [3]. In MS, this can go some way to meeting the need for information on disease characteristics, treatment behaviours, and healthcare availability for ‘real’ patients with the disease. Closer to product launch, RWE can provide further insights into early clinical experience, safety, resource use, patient tolerability, and identification of untreated patients [3].

MS is a lifelong disease that can span more than 40 years. The short duration of RCTs provides limited information on MS disease course and long-term treatment effects. RCTs alone may be acceptable in acute neurological diseases like meningitis and stroke, where discrete endpoints, such as survival or post-acute fixed disability, can be swiftly measured. Conversely, for MS, the potential effects of a disease-modifying therapy (DMT) on disease progression can only be obtained by collecting information on patients treated in routine clinical practice over many years. The endpoints commonly used in phase 2 and 3 MS studies, such as inflammatory lesions and relapse rates, are important surrogate markers for predicting the anticipated long-term prevention of disability, and these can only be validated via RWE methodologies.

## Collection of high-quality real-world data (RWD) in MS

Regardless of disease area, collection of high-quality datasets in clinical practice requires agreement on a common minimum dataset and the use of special documentation software (or modifications to existing electronic medical records) to collect demographic and disease-specific information. These standards are important to offset the perception that the quality of RWD is inferior to that gathered in sophisticated, modern-day RCTs. Electronic medical record-based data collection is usually event based, with visits and other data being recorded as they occur, whereas RCTs use a schedule design where the timing and data collection at each visit are explicitly specified. In MS, event-based reporting currently predominates (e.g. the Swedish MS registry (SMSreg), the European Database for Multiple Sclerosis system, and the iMED software used for data entry into the MS dataBase (MSBase)), although this is governed by specified minimum dataset descriptions. In contrast, the Multiple Sclerosis Documentation System 3D software used in multiple long-term follow-up studies imposes a defined visit schedule and is used predominantly in Germany, where it combines a ‘trial-like’ data documentation system with patient management [4]. Table 1 summarizes common sources of RWD [5] and gives examples of their use in RWE studies in MS [6–20].

**Table 1:** Methodologies for collecting real-world data [4] and examples of their application to multiple sclerosis (MS) studies

Source	Explanation	Advantages	Limitations	Examples in MS
RCTs extensions	• Supplement trial data	• Extend RCT into real world	• Short study duration • Limited patient population • No information on rare events	• ENDORSE: EQ-5D and SF-36 in patients treated with DMF [10]
Registries	• Population-based collection of information	• Long-term data natural history and disease management • Regional comparisons	• Non-randomized design • Incomparable patient groupings • Discontinuous visit schedule • Varying practice patterns • Inter-regional extrapolation not always appropriate	• Lyons MS database: disability progression [11] • SMSreg: epidemiology and treatment outcomes [16, 19] • MSBase: treatment outcomes (69 countries) [13–15, 17]
Prospective observational studies	• Pre-defined outcome measures in clinical practice	• Robust dataset powered to answer specific questions	• Hawthorne effect (patients behave differently because they know they are being observed)	• PANGAEA, TOP: fingolimod and natalizumab clinical trial [9, 20]
PASSs	• Voluntary or imposed by regulatory authorities for approval	• Ongoing monitoring of the benefit–risk profile	• No obligation for regulatory submission of protocols and study reports for voluntary PASSs	• PANGAEA: German voluntary PASS [20]
Administrative data	• Data required for reimbursement	• Quick, low-cost analyses • Large patient populations provide information on rare events	• Privacy concerns limit access to data • Incomplete or inaccurate data • Costs and charges are not differentiated	• Pharmetrics Plus™ and Medco databases: relapses, treatment compliance, resource use and inpatient stays [6–8]
Health surveys	• Descriptive data	• Provide broadly generalizable data	• Not product-specific • Subjective • Relies on participant recollection	• NARCOMS survey: symptoms, comorbidities and health-related quality of life [18]
EMRs	• Real-time data collection	• Low cost • Detailed information over long periods	• High-end statistical analysis tools required	• EMRs: diagnosis, disease progression, symptoms and treatment [12]

DMF, Dimethyl fumarate; EMR, Electronic medical record; ENDORSE, BG00012 monotherapy safety and efficacy extension study in MS; EQ-5D, European Quality of Life-5 dimensions questionnaire; MSBase, Multiple Sclerosis dataBase; NARCOMS, North American Research Committee on Multiple Sclerosis; PANGAEA, Post-Authorization Noninterventional German sAfety of GilEnyA in RRMS patients; PASS, Post-authorization safety study; RCT, Randomized controlled trial; SF-36, 36-Item Short Form; SMSreg, Swedish MS registry; TOP, TYSABRI Observational Program

## Performing and identifying high-quality RWE studies in MS

In MS, RWD is increasingly used for comparison studies to examine therapy choice and sequencing decisions. Outcomes are similar to those in RCTs and include relapse and disability progression rates, adverse events and therapy discontinuation events. Identification and mitigation of biases and careful consideration of study power are key factors for designing appropriate RWE studies. Indeed, various biases exist and require careful consideration in selecting appropriate comparators, patient populations, data sources, outcomes and statistical analyses [21].

Selected analytical techniques (e.g. regression and stratification) can reduce the bias introduced by non-randomized study designs. Regression can improve the accuracy of an estimated treatment effect on a particular outcome measure by adjusting the association between treatment and outcome to account for other variables that could affect said outcome. In MS, these variables include baseline Expanded Disability Status Scale (EDSS) score, prior relapse rate and disease duration. The type of regression model used largely depends on the outcome being measured [22]. Logistic regression is typically used for binary outcome measures (e.g. event occurred: yes/no), while proportional hazard estimates are used for continuous outcome measures (e.g. time to first relapse) [22]. Importantly, regression analysis uses all available data from the full patient cohort, enabling good statistical power, although it assumes that similar effects of an intervention occur across all subgroups and requires extrapolation when calculating estimates [22].

Stratification, whereby patient cohorts are divided into subgroups with similar variables, enables comparison of outcomes among patients with similar characteristics [22]. However, the potential for imbalance in other baseline covariates [22] requires the careful generation of statistically robust results, and sample sizes are necessarily smaller than for regression, reducing statistical power [22].

In many circumstances, propensity scoring has proven effective at reducing bias and is being used increasingly in longitudinal MS observational studies. In studies comparing the effect of two treatments, propensity scoring involves classification of the relationship between treatment assignment and baseline characteristics. Factors found to be different between treatment groups and associated with treatment choice are weighted to calculate the probability of any subject in the cohort being assigned a particular treatment – this is termed the ‘propensity score’. Subjects are then matched by the propensity score for comparison across treatment groups [23]. In effect, ‘unmatchable’ subjects, in whom a particular set of baseline characteristics leads to non-overlapping treatment assignment within a population, are removed from the outcome analysis and only ‘matchable’ subgroup outcomes are reported. Propensity scores can be used to aid stratification or regression through posterior adjustment of results [23]. When there are fewer than eight events per confounder, propensity scoring has been found to be a more robust method of eliminating bias than regression [24].

The introduction of bias through lack of randomization and blinding, as well as other methodological limitations of RWE studies, has raised questions about the validity of the evidence produced [25]. With this in mind, efforts have been made to promote good research practice and to advise researchers designing RWE studies to maximize the usefulness of the results obtained [21]. For cohort studies in general, checklists have been produced for both RCTs (CONSORT) [26] and for more general cohort studies (STROBE) [27]. Specifically for RWE studies, Dreyer et al. [28] recently compiled the Good Research for Comparative Effectiveness checklist to allow identification of RWE studies sufficiently high in quality for use in decision-making. Box 2 summarizes this checklist and the 10 ‘golden rules’ for identifying high-quality RWE studies, all of which are relevant to study design in MS.

## How has RWE helped in understanding the disease course and patient management in MS?

RWE generated from quality registries and other databases has greatly contributed to our understanding of MS disease course and risk factors. RWE studies have reported a decreased life expectancy for patients with MS compared with the general population [29], and have shown how factors such as increased age at disease onset and the primary progressive subtype of MS are associated with faster disability progression [30, 31]. They have also shown that, despite high variability in individual patients following conversion to secondary progressive MS and in patients with primary progressive MS, the mean or median progression rates between these disease subtypes are similar [31]. Other RWE studies have indicated a lower familial risk for developing MS than previously predicted [19], as well as an influence of race on outcomes [32].

RWE has also helped to guide patient management and treatment decisions in MS by answering questions related to treatment effects in clinical practice, which RCTs are unable to address. These effects are discussed in turn in the following sections.

### Comparative effectiveness of DMTs in clinical practice

In the RWE setting, comparative effectiveness of DMTs can be determined from registry data. In MS, web-based registries, such as MSBase and SMSreg, can provide this type of information at global and regional levels [16, 33]. For instance, data from SMSreg have shown that fingolimod treatment initiation is associated with stable EDSS scores and improvement in relapse rates, disease severity, cognition, and quality of life after 12 months [16]. MSBase data analyses have also demonstrated the positive effect of DMT treatment on first confirmed disability progression [34] and have been used extensively for comparative effectiveness studies to show that, in patients who relapse on ‘platform’ injectable DMTs, switching to fingolimod or natalizumab, rather than between injectable DMTs, can lead to improvements in time to first relapse, relapse rate, and disability progression and regression events [17, 35]. Interestingly, after relapse on platform therapy, a comparison of switch to fingolimod versus natalizumab indicated that switching to natalizumab from baseline therapies reduced relapse rates and increased sustained disability regression events more than switching to fingolimod, but that there was no difference in the rate of confirmed progression events between these treatments [14]. MSBase registry data also confirmed that relapse rates in patients switching to fingolimod from natalizumab were comparable to those switching from other therapies, and that an ideal treatment gap between these therapies was less than 8 weeks to reduce risk of early relapse [13] – a finding subsequently confirmed in an RCT [36]. MSBase data have also demonstrated variations between different baseline therapies, with patients treated with glatiramer acetate or subcutaneous interferon β-1α experiencing fewer relapses than those taking other baseline therapies [15]. Importantly, MSBase analyses (using propensity score matching) have generally replicated RCT results where known and align well with clinician experience, suggesting that biases are properly addressed using this dataset and statistical methodology.

### Safety and tolerability of a DMT in clinical practice

Registries are also a good source of information on safety and tolerability of a product in a real-world setting. The Immunomodulation and Multiple Sclerosis Epidemiology studies used SMSreg data to show that, although fingolimod and natalizumab are both well tolerated, fingolimod tolerability is reduced compared with that of natalizumab, especially in patients switching from natalizumab [37]. The Post-Authorization Noninterventional German sAfety of GilEnyA in RRMS patients (PANGAEA) study is a prospective, observational, registry-based study designed to collect data on effectiveness and adverse events in fingolimod-treated patients in standard clinical practice [20]. Prospective observational studies performed without registry data also provide an insight into the safety and tolerability of MS treatments in the real world, albeit on a smaller scale. The Safety, Tolerability and Adherence with Rebif^©^ study and Tysabri Observational Program interim analysis, for example, demonstrated that the safety profiles of subcutaneous interferon β-1α and natalizumab in relapsing-remitting MS patients in clinical practice are comparable with those in RCTs [9, 38].

### Impact of a DMT on resource use and medical costs

Claims databases provide valuable data on the impact of DMTs on resource use and medical costs, such as use of other medications and hospital stays. These pharmacoeconomic considerations are important influencing factors in formulary decisions. IMS PharMetrics Plus™ is a medical and pharmacy claims database containing the records of over 100,000 patients with MS across the USA [7, 8]. This dataset has been used to distinguish the effects of different DMTs on outcome measures such as adherence, persistence, inpatient stays and corticosteroid use for relapses [7, 8].

### Impact of a DMT on pharmacoeconomic outcomes

Pharmacoeconomic studies provide an insight into the cost-effectiveness of a DMT, including the direct effect of medication costs and indirect costs from health-related work absences or caregiver time. The PANGAEA and ProspEctive phArmacoeconomic cohoRt evaLuation (PEARL) registry studies of fingolimod- and injectable DMT-treated patients collect data on sick leave, hospitalization and physician consultations, and show that fingolimod treatment results in better pharmacoeconomic outcomes compared with baseline therapies [39]. Similar results have been observed with natalizumab treatment in patients in the USA through analysis of administrative claims databases [40].

### Impact of a DMT on patient-reported outcomes (PROs)

Patient-generated data can be used to assess the perceived clinical benefits of a therapy and is useful for examining other aspects of patient experiences with DMTs. Some RCTs, such as the Evaluate Patient Outcomes study [41], which assessed treatment satisfaction in patients treated with fingolimod or injectable DMTs, collect patient-generated data. Some observational RWE studies also assess PROs. PANGAEA and PEARL were designed to capture patient experience on effectiveness, tolerability [20] and treatment satisfaction, as well as on ease and convenience of taking their DMT as instructed [20, 42]. Additional PROs on quality of life, physical disability, cognition and fatigue can be obtained through health surveys [43].

## Evolving RWD collection in MS

Many countries across the world now collect MS RWD in registries and other databases [30, 44] (Table 2), and sharing of this information is critical to increase statistical power and to enable inter-regional knowledge transfer. Collaborations between MS registries, such as the European Register for Multiple Sclerosis, which includes data from 13 registries in Europe [45], further highlight the importance of information-sharing and learning on an international level by providing expanded datasets that allow cross-border analysis, interpretation and dissemination of results. Another collaborative example is the BigMS collaboration between MSBase and registries in France, Italy, Sweden and Denmark, which contains quality longitudinal datasets for over 130,000 patients with MS, with the first joint analyses planned for 2016. However, while the sharing of registry data sounds like an obvious positive step, in some cases this is not possible because it may breach the original patient consent. In these cases, efforts should be made to either re-contact the patients or to strip out all patient identifiers before the data are shared. Certainly, with the modem age of big data and the need to combine datasets to improve statistical power, consent to share data ought to be included in the consent forms for any new programmes.

**Table 2:** Key multiple sclerosis (MS) registry studies in Europe, North America and globally

Registry	Patients, n
*Europe*	
Croatia (SDMSH)	2477
Denmark (DMSC)	12,500
France (EDMUS)	~40,000
Germany (DMSG)	~30,000
Germany (MSDS3D Users)	>5000
Greece (GMSS)	3500
Italy (iMed/web)	~20,000
Norway (Nasjonal kompetansetjeneste for multippel sklerose – MS)	5100
Russia	21,500
Serbia	3500
Spain (Catalonia; EpidEMcat)	>5000
Sweden (SMSreg)	12,900
Switzerland (SMSR)	270
United Kingdom (MS Register)	8300
*North America*	
Canada (London Ontario)	1099
Canada (British Columbia)	2837
USA (New York; NYSMSC)	9600^a^
USA (NARCOMS)	19,297
*Global*	
MSBase (31 countries)	39,030

Patient numbers are derived from the original publications [30, 44] and may have changed

^a^Numbers updated by NYSMSC, 6 May 2016

DMSC, Danish Multiple Sclerosis Center; DMSG, National Multiple Sclerosis Society of Germany; EDMUS, European Database for Multiple Sclerosis; EpidEMcat, Catalonia MS Registry; GMSS, Greek Multiple Sclerosis Society; NARCOMS, North American Research Committee on Multiple Sclerosis; NYSMSC, New York State Multiple Sclerosis Consortium; MSBase, Multiple Sclerosis database; SDMSH, Croatian Multiple Sclerosis Society; SMSR, Scottish Multiple Sclerosis Register; SMSreg, Swedish MS registry

MS RWD can be collected within registries that contain information on other disorders that can be used to inform neurologists and improve patient management across a range of neurologic diseases. The Swedish Neuro Registries includes MS RWD from SMSreg as well as other disease-specific data, including Parkinson’s disease, epilepsy and myasthenia gravis. However, disease specificity and usability of registry data is fundamentally assured by the registry participant’s agreement to collect a uniformly defined ‘minimum dataset’ at a relatively set frequency. This is a major limitation of MS registries, and if data are to become more reliable and powerful, then standards for data input are needed and these need to be audited with validated quality control checks.

Although registries currently collect information on a variety of different clinical, pharmacoeconomic and safety outcome measures, the quality of magnetic resonance imaging (MRI) data in MS registries is currently limited. The descriptive, semi-quantitative MRI T2 lesion measures that are often recorded (e.g. Barkhof–Tintoré criteria) capture the severity of cerebral and spinal cord MRI abnormalities in MS relatively poorly, although they do provide a broad characterisation of lesion load and location.

The argument for inclusion of better, standardised MRI data in registries, and indeed their acquisition in clinical practice, is strong. MRI provides evidence of disease activity in MS [46] and new T2 hyperintense and T1 hypointense lesion development, particularly whilst on treatment, correlates with long-term disability progression [47, 48]. The increased rate of brain volume (BV) loss in patients with MS compared with healthy controls [49], indicating myelin, axonal and neuronal loss [50], is associated with cognitive impairment and disability in MS [51–53]. As BV loss is differentially affected by different MS therapies, standardized measures of BV change in clinical practice captured in real-world registries could provide information on the relative efficacy of MS therapies [54], with such information warranting inclusion in prognostic models. Inclusion of quantitative baseline MRI parameters would also improve patient matching to compare different treatment switch decisions in long-term RWE studies. We believe that a technological solution is imminent. Indeed, third party providers performing online automated MRI analytics (e.g. IcoMetrix, NeuroQuant) are emerging and have achieved medical device registration for use in clinical practice. The MRI manufacturers themselves are also developing automated lesion and BV algorithms that will likely be incorporated in standard MRI analytical packages within the next 2 years.

Importantly, RWE collection systems are becoming increasingly flexible, with the ability to exchange data with different sources (e.g. physician-, MRI- and patient-generated data) [55]. Additionally, key providers of RWD collection systems understand the need for creating added value for contributing clinicians, with features like graphical outcomes recording, own data exportation and data benchmarking analysis being offered through the platforms.

## Conclusions

RWE provides a valuable contribution to the evidence base for the use of MS therapies by supplementing RCT data and providing information on long-term effectiveness and tolerability of treatments in a real-world setting across generalizable populations. It provides longitudinal outcome information that can be directly used for pharmacoeconomic evaluation and examination of treatment patterns and sequencing outcomes. Robust study designs, including appropriate data collection and analytical methods as well as a unified minimum dataset, are critical for generating evidence that can be reasonably used to influence treatment decisions and guidelines, and satisfy the growing need of stakeholders to monitor DMT performance post-approval.

RWE generation can be further expanded in MS, as some important outcome measures are not routinely collected in real-world databases. By collecting information on additional outcome measures, such as MRI and cognition, their importance in guiding treatment could be examined. By continually expanding the data pool through collaborations, the validity and utility of RWE to physicians and regulatory bodies will be improved, fostering greater and better physician/patient engagement.

## Box 1. About real-world evidence (RWE) studies [1, 5]

Randomised controlled trials (RCTs) are the ‘gold standard’ in the generation of efficacy and safety evidence of a product in restricted trial settings.*Advantages* 1. Can mimic RCTs in the real world to allow assessment of a drug in the clinical setting2. Complement the evidence base from RCTs by assessing a diverse range of outcome measures to provide information that may not be captured by other means, including:• comparative effectiveness data between multiple therapies• information on long-term disability outcomes• better characterization of long-term exposure risks and benefit–risk profiles• patient-reported outcomes• economic outcomes*Challenges* 1. They cannot address the potential benefits and risks of a product in the real world [1, 5] because enrolment is restricted by disease-activity criteria, and subjects with comorbid conditions are typically excluded2. Active comparator arms may not reflect the usual standard of care and selected endpoints can be artificial or chosen to maximize statistical power, thereby limiting the real-world relevance of study conclusions3. There are significant ethical concerns about conducting placebo-controlled trials in countries where disease-modifying therapies are approved and reimbursed [2]; these pivotal trials are therefore conducted in countries without these approvals, and thus in populations different to potential target markets4. There are many potential biases in real-world treatment comparisons and variations in data quality caused by acquisition in busy clinics

## Box 2. The ten golden rules for identifying high-quality real-world evidence (RWE) studies [28]

**Rules for identifying high-quality RWE studies1. Treatment details and primary outcomes should be adequately recorded** • Sufficient detail should be recorded so that information on treatment, including dose, regimen and mode of administration, can be determined• Enough meaningful and robust information to allow primary outcomes of the study to be established**2. Primary outcomes should be measured objectively**• Objective primary outcome measures help to reduce bias between datasets• Objective primary outcome measures for multiple sclerosis (MS) would include the number and volume of T2 lesions and disability progression as measured by the Expanded Disability Status Scale (EDSS)**3. Primary outcomes should be valid** [28]• The primary outcomes for the study should be appropriate for the research question to be answered (e.g. EDSS progression and relapse rate in MS, and claims data for resource use)**4. Primary outcomes should be identified and measured equally in treatment and comparison groups**• Data, tools and methods used for evaluating primary outcomes should be identified and measured in the same way in both the treatment and the comparison groups• Comparator arms should be clinically relevant [1]**5. Confounders of treatment effect should be recorded**• Any variables that could potentially impact the primary outcome of the study should be recorded (e.g. in MS, age of onset of disease, number of relapses in the year prior to start of the study, EDSS score at entry into study, previous treatment with disease-modifying therapies, and number of lesions at baseline)**6. The study population should be restricted to new users of the treatment being assessed**• Include only treatment-naïve patients to enable the frequency of early treatment effects to be determined and to limit bias associated with delayed treatment or loss of effectiveness• If treatment naivety cannot be achieved, then washout should be considered**7. Data should be collected for the same time period for treatment and comparison groups**• Data from patients in treatment and comparator groups should be from within the same time period to ensure that the same standard of care is applied to both groups and that time-dependent effects are minimised• Historical data may be used in some cases (e.g. when comparing with older treatments that are no longer widely used)**8. Confounders of treatment effect should be taken into account in the study design and analysis**• Studies should either restrict patient inclusion or employ analytical methods such as propensity scoring, stratification and regression to control for confounding factors• Methods used for reducing bias should be properly reported; however, it is still important to be aware of the limitations of the statistical techniques used to reduce bias as they can lead to an inaccurate estimation of the effect of a treatment [1]**9. The study design should take into account ‘immortal time bias’**• Study design should ensure that any events that occur before treatment exposure (in ‘immortal time’) are not included in the analysis, as this can exaggerate drug benefits**10. Key assumptions on which primary outcomes are based should be analyzed**• Assumptions should be analysed to verify their validity and reduce bias

## Abbreviations

BV: brain volume
DMT: disease-modifying therapy
EDSS: Expanded Disability Status Scale
MRI: magnetic resonance imaging
MS: multiple sclerosis
MSBase: multiple sclerosis database
PANGAEA: Post-Authorization Noninterventional German sAfety of GilEnyA in RRMS patients
PEARL: prospEctive phArmacoeconomic cohoRt evaluation
PRO: patient-reported outcome
RCT: randomized controlled trial
RWD: real-world data
RWE: real-world evidence
SMSreg: Swedish MS registry
